# Recent Understanding and Future Directions of Recurrent Corticotroph Tumors

**DOI:** 10.3389/fendo.2021.657382

**Published:** 2021-04-26

**Authors:** José Miguel Hinojosa-Amaya, César Ernesto Lam-Chung, Daniel Cuevas-Ramos

**Affiliations:** ^1^ Pituitary Clinic, Endocrinology Division, Department of Medicine, Hospital Universitario “Dr. José E. González” UANL, Monterrey, Mexico; ^2^ Neuroendocrinology Clinic, Department of Endocrinology and Metabolism, Instituto Nacional de Ciencias Médicas y Nutrición Salvador Zubirán, Mexico City, Mexico

**Keywords:** ACTH, corticotropin, Cushing disease, cortisol, molecular biology, corticotroph tumor, pituitary tumor

## Abstract

Corticotroph tumors (CTs) are pituitary neoplasms arising from the Tpit lineage, which may or not express adrenocorticotrophic hormone (ACTH). Functioning CTs cause Cushing’s disease (CD), which has high morbidity and mortality due to hypercortisolemia. “Non-functioning” or silent CTs (SCT) and the Crooke’s cell subtypes do not cause CD and may be asymptomatic until manifested by compressive symptoms and are more frequently found as macroadenoma. Both tend toward more aggressive behavior, recurrence, and a higher rate of malignant transformation to pituitary carcinoma. Tumorigenesis involves genetic, epigenetic, and post-transcriptional disruption of cell-cycle regulators, which increase cell proliferation, *POMC* overexpression, ACTH transcription, and/or hypersecretion. Furthermore, functioning CTs develop resistance to glucocorticoid-mediated negative feedback on ACTH secretion, through increased expression of testicular orphan nuclear receptor 4 (TR4), heat-shock protein 90 (HSP90), and loss-of-function mutation of CDK5 and ABL enzyme substrate 1 (*CABLES1*) gene. Overt autonomous hypercortisolemia is difficult to control, and multiple diagnostic studies and therapeutic modalities are commonly required. Cell-cycle regulation depends mainly on p27, cyclin E, cyclin-dependent kinases (CDKs), and the retinoblastoma protein (Rb)/E2F1 transcription factor complex. Gain-of-function mutations of ubiquitin-specific protease (*USP*) 8, *USP48*, and *BRAF* genes may subsequently cause overexpression of epithelial growth factor receptor (EGFR), and enhance POMC transcription, cell proliferation, and tumor growth. Epigenetic changes through micro RNAs and decreased DNA deacetylation by histone deacetylase type 2 (HDAC2), may also affect tumor growth. All the former mechanisms may become interesting therapeutic targets for CTs, aside from temozolomide, currently used for aggressive tumors. Potential therapeutic agents are EGFR inhibitors such as gefitinib and lapatinib, the purine analog R-roscovitine by dissociation of CDK2/Cyclin E complex, the HSP90 inhibitor silibinin (novobiocin), to reduce resistance to glucocorticoid-mediated negative feedback, and BRAF inhibitors vemurafenib and dabrafenib in *BRAF V600E* positive tumors. This review summarizes the molecular mechanisms related to CTs tumorigenesis, their diagnostic approach, and provides an update of the potential novel therapies, from the lab bench to the clinical translation.

## Introduction

Cushing’s disease (CD) is caused by oversecretion of adrenocorticotropic hormone (ACTH) by a pituitary corticotroph adenoma and is the most frequent cause of endogenous Cushing’s syndrome (CS) (1). This rare disease is usually diagnosed between the fourth and fifth decade of life, is more frequent in women, and accounts for 4 to 8% of all pituitary tumors (2). Most corticotroph tumors are sporadic and less than 5% of these have been related to familial diseases (3). Endocrine hereditary syndromes with CD include multiple endocrine neoplasia (MEN) type 1 (4), familial isolated pituitary adenomas (FIPAs) (5), Carney complex (6), and DICER1 syndrome (3, 4). A gain-of-function mutation in the ubiquitin-specific protease 8 (USP8) gene has been associated with sporadic CTs in about 20 to 60% of patients (7–10).

On the other hand, silent corticotroph adenomas (SCTs) do not cause hypercortisolism or CS but their recurrence rate reaches 36%, and up to 18% will recur multiple times, being this subtype more aggressive than other pituitary tumors (11). Moreover, pituitary carcinomas frequently arise from the corticotroph lineage (12).

CTs emerge from complex mechanisms that involve cell-cycle dysregulation, genomic abnormalities, and others that currently are not completely understood. There is also controversy on the definitions of remission and recurrence, especially in SCTs.

This manuscript summarizes the pathological mechanisms related to corticotroph tumorigenesis which are implicated in ACTH secretion or clinical silence, recurrence, and aggressive behavior, and provides a review on the potential novel therapies which may target both silent and functioning CTs.

## 2017 WHO Classification for Pituitary Tumors

A corticotroph tumor is currently defined as a Tpit-positive neoplasm either if ACTH-positive or not. Most corticotroph tumors have ACTH immunoreactivity and cause ACTH-dependent CS if functioning, known as Cushing’s disease (CD), or to be clinically non-functioning (silent) corticotroph tumor (SCT) (11, 13).

SCTs were defined in 1970 as ACTH-positive staining pituitary tumors which do not cause evident hypercortisolism or CD (14). The expected prevalence is 4.8 to 6.8% among adult non-functioning pituitary adenomas (11) and has been reported only once in the pediatric population (15). Diagnosis is made in a retrospective fashion with histopathological staining since clinical factors and presurgical laboratory tests cannot discern them from other silent adenomas (16). One retrospective study suggested female sex, cavernous sinus invasion, intra-tumoral hemorrhage on MRI, and decreased ACTH response to hypoglycemia, may be associated with SCTs (17). Silent and functioning corticotroph tumors can be further divided into sparsely granulated, densely granulated, and Crooke cell adenomas (13, 18). The current classification recommends against the term “atypical adenoma” and instead proposes routine use of tumor proliferation markers, clinical parameters such as tumor invasion, and evaluation of tumor types that may be more clinically aggressive. Interestingly, two adenoma subtypes which commonly show aggressive behavior fall into the corticotroph lineage: the SCTs and the Crooke’s cell adenomas (19, 20).

A clinicopathological classification have been proposed to predict disease-free and recurrence/progression-free status of pituitary tumors in order to guide clinicians to choose the best therapy (21).

## Tumorigenesis on Functioning Corticotroph Tumors

### Cell Cycle Division

**Figure 1:**
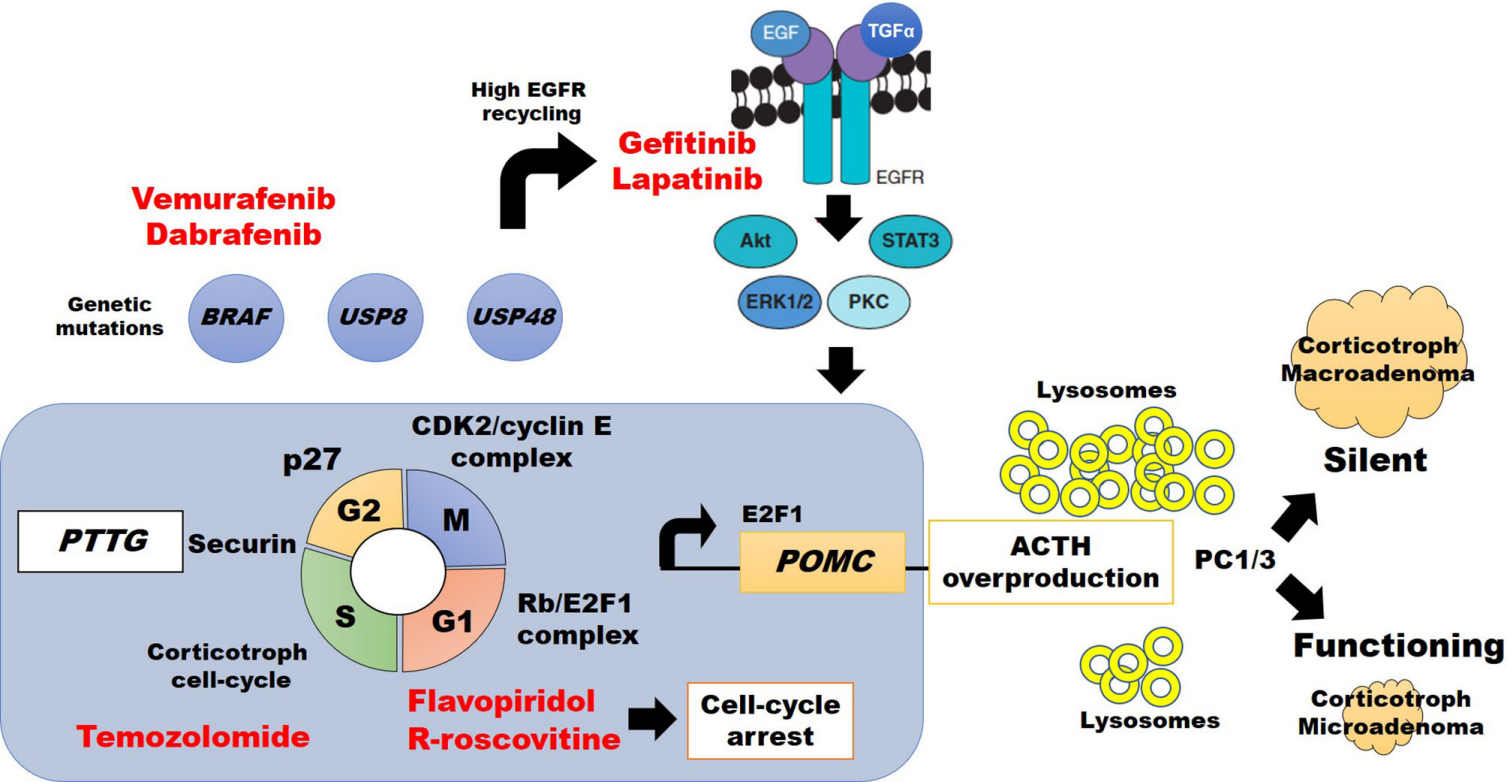
Abdnomal regulation of cell cycle division, ACTH synthesis, and potential therapeutic approach (highlighted in red) in silent vs. clinically active corticotroph tumors (see text for details).

Progression of the cell cycle division in pituitary cells is regulated by the pituitary tumor-transforming gene (PTTG) which encodes a securin protein, which in turn regulates activity in the G1/S phase, influences chromosomal stability, and tumorigenic activity (**Figure 1**) (22, 23). Such tumorigenic activity is downregulated by proteins of the INK4 (p16, p15, p18, p19) and Cip/Kip (p21, p27, and p57) families (24, 25) to control cyclins’ and cyclin-dependent kinases’ (CDKs) activity (26). CDK4 and CDK6 are active during the G1 (gap 1) phase after association with cyclins D1, D2, and D3. Progression to S (synthesis) phase occurs after cyclin E1 and E2 activation. At this point, downregulation is driven by the INK4 family proteins. Then proteins from the Cip/Kip family, particularly p27, induce cyclin A1 and A2 on CDK1 and CDK2 activity to regulate the end of S phase and move through G2, and then induce CDK1/cyclin B1 and B2 to progress into the M (mitosis) phase (**Figure 1**).

Experimental treatment with the CDKs (1, 2, 4, 6, and 7) inhibitor flavopiridol in a double CDK4/p27 knockout mouse, resulted in tumor shrinkage by cell-cycle arrest at G1 and G2 phases, confirming the importance of CDKs on pituitary tumor growth (27). Consistent with these findings, the second-generation CDK1 and CDK2 inhibitor R-roscovitine (seliciclib), inhibiting the association of CDK2/cyclin E complexes (28), caused a reduction of POMC promoter expression in ~40% in a double transgenic POMC : PTTG zebrafish model, and in murine AtT20 corticotroph adenoma models (29–31) Other studies also found murine p27 knock-out was associated with enlarging corticotroph tumors within 12 months (32–34), and molecular research of recurrent human corticotroph adenomas or carcinomas showed lower or absent p27 staining (35). Therefore, CDKs, cyclin E, and p27 are key molecules that modify CTs’ behavior. Also, increased expression of cyclin E downregulates the tumor suppressor Brahma-related gen 1 (Brg1), which has synergic action with glucocorticoid receptor (GR), orphan nuclear receptor growth factor 1B, and histone deacetylase 1 (HDAC1) to decrease the POMC promoter expression. Disruption of this complex by cyclin E may increase POMC and ACTH synthesis and, as consequence, CD (36).

### Rb Protein

Dysfunction of the Rb protein, a tumor suppressor regulator, was first described in retinoblastoma tumor cells and then, in heterozygous or homozygous Rb knockout mouse models were identified the higher risk of pituitary tumors development (37–39). Rb protein is involved in cell-cycle regulation of many tumors, including those of the pituitary (22, 23, 37, 38, 40, 41). However, the role of Rb protein in human CTs seems to be different than in mice models because patients with familial retinoblastoma do not always harbor pituitary tumors, and CTs have been rarely reported (39, 42). Human Rb dysfunction is more commonly associated with aggressive macroadenomas or corticotroph carcinomas (43, 44). It is also a negative cell-cycle regulator that controls G1/S progression by inhibition of the corticotroph-specific E2F1 transcription factor (**Figure 1**) (45–47).

### E2F Family Proteins

The proteins of the E2F family (E2F1 to E2F8) are associated with tumorigenesis of multiple cell lines. Regarding CS, the Rb/E2F1 complex formation has been described both in pituitary corticotroph tumors (CD) (48), and ectopic ACTH-secreting carcinomas (29). Araki and colleagues also reported E2F1 binding directly to *POMC* promoter, increasing its transcription and ACTH synthesis (29, 49). An E2F inhibitor (HLM006474) showed dose-dependent suppression over POMC mRNA expression on CT (45). Therefore, the Rb/E2F1 complex regulates the progression from G1 to S phase of the cell cycle, and free E2F1 acts directly over the *POMC* promoter (**Figure 1**) (29, 45). Further research is needed to define whether E2F1 *per se* has a relationship with corticotroph tumor behavior.

### Tpit

Tpit (formerly known as TBX19) together with Pitx1 interacts on their specific response element sequences at the *POMC* promoter increasing expression and synthesis of POMC which, after post-transcriptional processing and multiple sites of enzyme sliding, is converted into six main proteins including ACTH (50). When Tpit is expressed in normal corticotroph cells, cyclin E is no longer detected (24). Murine and zebrafish corticotroph tumor models found an abnormal cyclin E upregulation within the neoplastic tissue, causing abnormal reentry to cell-cycle division and centrosome instability, promoting tumorigenesis (30, 36, 51). Interestingly, R-roscovitine, which also targets the Tpit binding region (TCACACC) of the POMC promoter, interferes with cyclin E and E2F1 and causes suppression of ACTH synthesis and secretion in a dose-dependent manner (31). This suppression of the POMC promoter and ACTH release by R-roscovitine was also confirmed in ectopic ACTH-secreting tumors (29, 52). A phase II clinical trial to evaluate R-roscovitine in patients with confirmed recurrent CD is currently closed with pending results (ClinicalTrials.gov NCT02160730).

## Genetic Mutations and Related Mechanisms for CTs Tumorigenesis

### Epidermal Growth Factor Receptor

Epidermal growth factor receptor (EGFR) is a tyrosine kinase receptor that is commonly expressed in normal corticotroph cells. EGFR is one of the most important inductors of POMC transcription, and ACTH synthesis (53), and EGFR overexpression was found in CT of transgenic murine models, causing CD (45). EGFR signalizes by phosphorylating the extracellular response kinase 1/2 (pErk1/2) and this pathway induces POMC transcription through a higher expression of free E2F1. The mechanisms by which *POMC* transcription is induced by EGFR are not completely understood (8, 9).

### USP8

USP8 is an enzyme that mediates the deubiquitination of intracellular vesicles of normal corticotroph cells and therefore avoids lysosomal degradation. Gain-of-function mutations in the *USP8* gene lead to an impaired association with 14-3-3 protein, resulting in increased deubiquitination of endocytosed vesicles, including EGFR (8, 54, 55). Such deubiquitination causes EGFR recycling and overexpression. The action of both EGF and transforming growth factor-alpha (TGF-alfa) on more abundant EGFR results in higher *POMC* expression and ACTH synthesis (**Figure 1**) (8, 9). Interestingly, such USP8 mutation is specific in CTs and it was not identified in other types of pituitary tumors (10), was reported more frequently in women (8, 9, 56), and was identified in patients with higher cortisol levels and more aggressive CTs (57). In contrast, a smaller sample (n=13) of silent, less aggressive, CTs showed no USP8 mutations (56).

USP8 mutations in tumors causing CD have a prevalence of 30 to 50%. Nevertheless, CD USP8-mutated tumors did not show a higher rate of tumor aggressive behavior, suggesting that complementary mechanisms need to be elucidated (8, 58, 59). (8, 9).

### USP48 and BRAF

Chen et al. reported recurrent mutations in the *USP48*, which predominantly encodes p.M4151 or p.M415V in 23% (21/91), and *BRAF*, encoding p.V600E, in 16% (15/91) patients with CD (60). *USP48* and *BRAF* mutants enhance POMC transcription, suggesting an additional mechanism for ACTH excess (60). Such results may also have therapeutic implications in the future, since BRAF inhibitors vemurafenib and dabrafenib are currently FDA approved for the treatment of late-stage melanoma (see below), and might be tested in patients with corticotroph cells harboring *BRAF V600E* in recurrent CD (60).

## Epigenetic Changes in CTs Tumorigenesis

Somatic mutations are present in a small proportion of patients with CD; therefore, epigenetic changes have been considered as a potential mechanism of tumorigenesis (61).

### Histone Deacetylase Type 2

Histone deacetylase type 2 (HDAC2) regulates gene expression by removing acetyl groups from lysine residues located at the N-terminal region of core histones. Inhibition of HDAC2 reduces the survival of normal corticotroph cells and may impair ACTH secretion (62). A lower expression of this enzyme has also been associated with the development of glucocorticoid resistance (36).

DNA acetylation and methylation are directly related to changes in p53 and Rb protein expression, which have an important role in tumor development and progression (61).

#### Micro RNA (miRs)

The miRs are small and non-coding RNAs that play an important role in a variety of cellular processes, including cell development, differentiation, and apoptosis. Binding miRs to specific messenger-RNAs (mRNA) may block their translation to proteins. Some miRs have been described in pituitary tumorigenesis (63–65) including CTs (66–68). More aggressive behavior of CTs is associated with overexpression of miR-25, miR-93, and miR106b (miR-106b~25 cluster) (67). A higher expression of the miR-106b~25 cluster has also been described in aggressive Crooke cell adenomas (67). Crooke cell adenomas are characterized by cytoplasmic hyaline inclusions instead of normal densely or sparsely granulation (20, 69). The triggering mechanisms causing the Crooke hyaline changes are currently unknown. Galectin 3 (*LGALS3*), which is regulated by miR-493, is also overexpressed in CTs and correlates to increased tumor aggressiveness (70). In contrast, a decreased expression of miR-141 has been found to predict a higher probability of CD remission (66). Unfortunately, the pathological expression of miRs in CTs has been reported in a small number of patients and further research is needed.

## Tumorigenesis of SCT

Compared to ACTH-secreting CTs, SCT may have a different embryologic origin arising from POMC-expressing cells in the pars intermedia of the pituitary gland (71). Lack of ACTH oversecretion from SCT has been related to two mechanism: The presence of a higher amount of lysosomes in the tumor’s cytoplasm, which causes premature destruction of ACTH before release (72); and disruption of POMC-product processing due to dysfunction or reduced expression of prohormone convertase 1/3 (PC1/3) resulting in an inability to synthesize mature ACTH molecules (**Figure 1**) (72, 73). PC1/3 cleaves POMC in dibasic sites comprising lysine (K) and arginine (R) resulting in pro-adrenocorticotropin (pro-ACTH) and then to ACTH (50). Interestingly, SCTs transforming to functional ACTH-secreting CTs show a higher PC1/3 expression (73). Increased prevalence of USP8 mutations has not been reported in SCT, suggesting different genetic or epigenetic backgrounds (56). However, some similarities in related etiological pathways may be found soon since SCTs have been reported to become functioning CTs and vice versa (73).

## Disruption of Negative Feedback by Glucocorticoids

**Figure 2:**
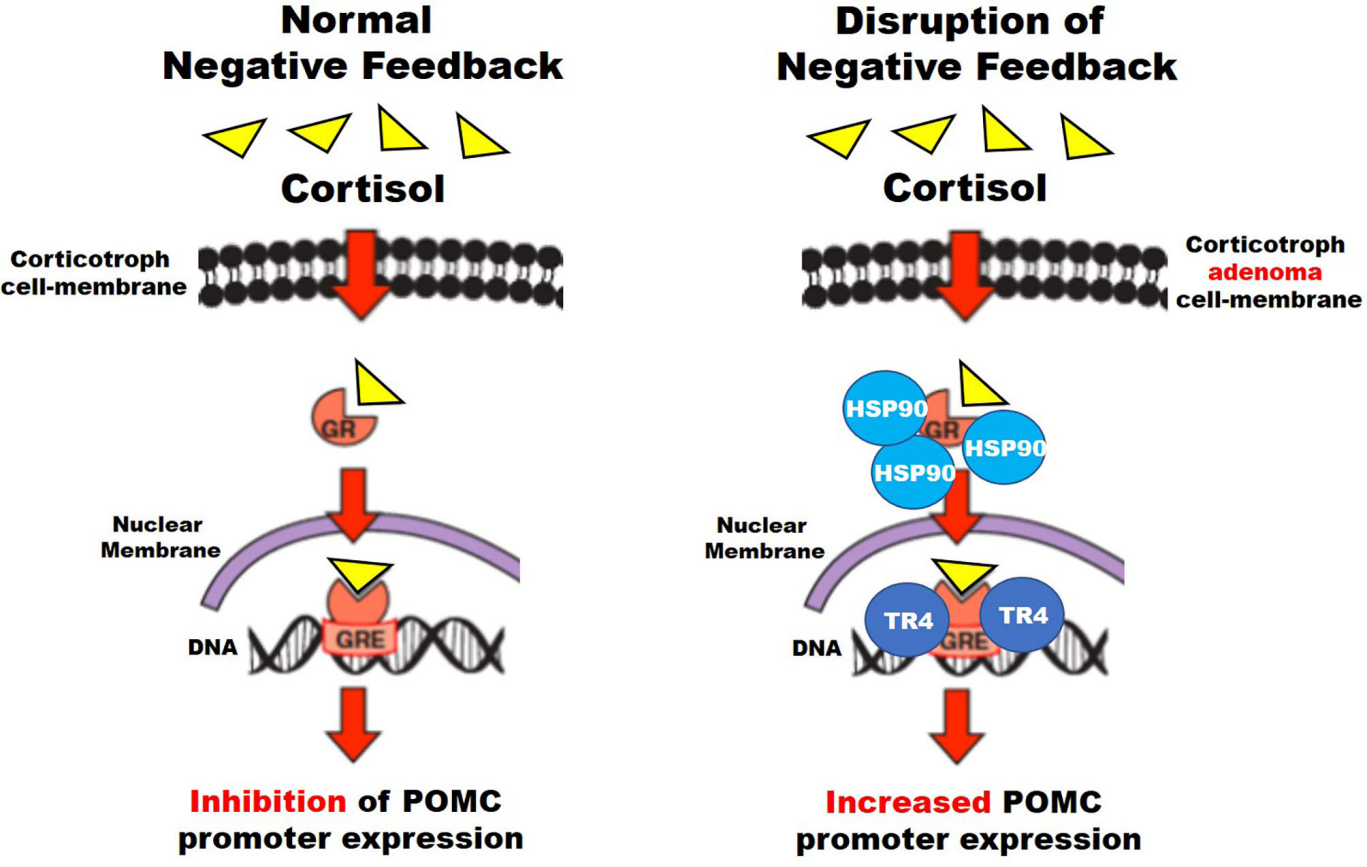
Suggested mechanisms for disruption of negative feedback in corticotroph tumors (see text for details).

Corticotroph glucocorticoid resistance and negative feedback disruption are common features of CD. Possible mechanisms have been related to USP8 mutations, testicular orphan nuclear receptor 4 (TR4), and heat shock protein 90 (HSP90) (**Figure 2**). TR4 overexpression is highly prevalent in CTs. This receptor increases *POMC* transcription, ACTH secretion, and tumor cell growth through the mitogen-activated protein kinase/extracellular signal-regulated kinase (MAPK/ERK) pathway (74, 75). TR4 directly inhibits GR’s interaction with the *POMC* promoter region and causes resistance to negative feedback in CD (**Figure 2**) (74, 75). Therapy targeting TR4 in CD may be evaluated in the future.

HSP90 is a chaperone protein that induces conformational changes of different proteins, including the GR. HSP90 overexpression causes an increased binding to the GRs preventing dissociation from the chaperone system and their translocation to the nucleus, which otherwise suppresses *POMC* transcription, and disrupts glucocorticoid-mediated negative feedback (76).

Glucocorticoid resistance also has been found in four CDK5 and ABL enzyme substrate 1 (*CABLES1*) gene germline missense variants in 4 female patients, and a loss-of-function mutation in 146 pediatric and 35 adult patients. All cases showed aggressive tumor behavior (5, 77).

## From the Lab Bench to the Patient With Recurrent Corticotroph Tumors

### Implications for Future Treatments

#### USP8 and EGFR Pathway

A USP8 inhibitor (9-ethyloxyimino-9H-ideno[1,2-b] pyrazine-2,3dicarbonile) has been used for *in vitro* studies and animal models but has not been studied for therapeutical purposes. In AtT20 cells, USP8 inhibition with this molecule downregulated EGFR expression, decreased POMC transcription and ACTH synthesis and secretion, and induced cell apoptosis (8, 54, 55, 59, 78). Future research may explore USPs as potential target molecules for the treatment of both silent and secreting corticotroph adenomas.

One phase II open-label clinical trial in patients with USP8-mutated corticotroph tumors and CD was registered in 2015 but data on results has not been updated ever since (https://clinicaltrials.gov/ct2/show/NCT02484755: Targeted Therapy With Gefitinib in Patients With USP8-mutated Cushing’s Disease) (54, 79, 80). Lapatinib is another tyrosine kinase inhibitor acting over the EGFR and HER2 receptors, which has been tested *in vitro* to decrease POMC mRNA and ACTH levels in murine AtT-20 corticotroph cells. It also decreased PTTG gene expression, cell proliferation, and induced apoptosis (**Figure 2**). Results were replicated *in vivo*, tested in murine animal models (54, 81) but not in humans.

#### USP48 and BRAF Inhibition

In the presence of a wild USP8 phenotype, whole-exome sequencing has discovered mutation on both *USP48*, which causes ACTH mRNA overexpression, and *BRAF* genes, which in turn upregulates Erk1/2 phosphorylation with subsequent Nurr77, c-jun, and c-fos activation leading to increased POMC transcription (60). BRAF inhibitors such as vemurafenib and dabrafenib have been used to treat BRAF V600 mutation-positive malignancies and have become a potential option to treat BRAF V600-mutated CD (**Figure 1**). One *in vitro* study using vemurafenib in BRAF V600-mutated corticotroph cells showed a reduction in ACTH secretion after 1-day incubation (82, 83).

#### Corticotroph HSP90 Inhibition

Silibinin is a HSP90 C-terminal inhibitor found in the milk thistle (*Silybum marianum*) previously used to treat amatoxin poisoning and studied as a potential treatment of many malignancies (prostate, breast, hepatic cell, lymphoblastic leukemia), showing an acceptable safety profile (84, 85). This compound releases GRs from HSP90, therefore reestablishing glucocorticoid-mediated negative feedback on ACTH secretion (**Figure 2**) (80). There are no ongoing clinical trials for the evaluation of silibinin for CD.

#### Cyclin-Dependent Kinases and Cyclin E/E2F1 Pathway Inhibition

Cyclin-dependent kinases (CDKs) are essential regulatory proteins of the cell cycle progression (86). Pituitary cyclin E/E2F1 is a potential molecular target of pituitary ACTH-dependent hypercortisolism. In corticotroph tumor AtT20 cells murine models, R-roscovitine down-regulates cyclin E/E2F1 resulting in suppressed POMC expression (30). Also, in human pituitary corticotroph tumors treated with R-roscovitine, resulted in inhibition of the kinase activity (31).

## Conclusions

Mechanisms of CTs tumorigenesis have identified hundreds of potential genes, miRs, proteins, and peptides which are up or downregulated in comparison to the normal anterior pituitary. These changes may represent potential targets for pharmacological treatment or have an impact on the prediction of CD and SCTs recurrence. Further research is needed to better understand CT origin and pathophysiology. This may also lead to the identification of novel markers of disease severity or progression, and diagnostic tests with higher performance to predict recurrent CD or SCT. Although novel molecular markers are still not clinically validated as predictors of recurrence in CT, some of them are currently targeted for research as potential novel therapies.

## Author Contributions

JH-A designed the review. JH-A, CL-C, and DC-R prepared the draft manuscript. DC-R reviewed and approved the submitted version. All authors contributed to the article and approved the submitted version.

## Conflict of Interest

The authors declare that the research was conducted in the absence of any commercial or financial relationships that could be construed as a potential conflict of interest.
